# Identification of the Binding Sites on Rab5 and p110beta Phosphatidylinositol 3-kinase

**DOI:** 10.1038/s41598-017-16029-6

**Published:** 2017-11-23

**Authors:** Dielle E. Whitecross, Deborah H. Anderson

**Affiliations:** 1 Cancer Research, Saskatchewan Cancer Agency, 107 Wiggins Road, Saskatoon, Saskatchewan S7N 5E5 Canada; 2 Departments of Oncology and Biochemistry, College of Medicine, 107 Wiggins Road, University of Saskatchewan, Saskatoon, Saskatchewan S7N 5E5 Canada

## Abstract

Rab5 is a small monomeric GTPase that mediates protein trafficking during endocytosis. Inactivation of Rab5 by GTP hydrolysis causes a conformational change that masks binding sites on its “switch regions” from downstream effectors. The p85 subunit of phosphatidylinositol 3-kinase (PI3K) is a GTPase activating protein (GAP) towards Rab5. Whereas p85 can bind with both Rab5-GTP and Rab5-GDP, the PI3K catalytic subunit p110β binds only Rab5-GTP, suggesting it interacts with the switch regions. Thus, the GAP functions of the catalytic arginine finger (from p85) and switch region stabilization (from p110β) may be provided by both proteins, acting together. To identify the Rab5 residues involved in binding p110β, residues in the Rab5 switch regions were mutated. A stabilized recombinant p110 protein, where the p85-iSH2 domain was fused to p110 (alpha or beta) was used in binding experiments. Eleven Rab5 mutants, including E80R and H83E, showed reduced p110β binding. The Rab5 binding site on p110β was also resolved through mutation of p110β in its Ras binding domain, and includes residues I234, E238 and Y244. This is a second region within p110β important for Rab5 binding. The Rab5-GTP:p110β interaction may be further elucidated through the characterization of these non-binding mutants in cells.

## Introduction

Class IA phosphatidylinositide 3-kinases (PI3Ks) play key roles in signaling from cell surface receptors through the generation of phosphatidylinositol 3,4,5-trisphosphate (PIP3) which activates the Akt/mTOR pathway^1–5^. Class IA PI3Ks are heterodimers consisting of a p110 catalytic subunit stabilized by a p85 regulatory subunit^6^. There are three isoforms of p110 designated as class IA: p110α, p110β, and p110δ^7^, of which p110β has some unique properties. In addition to its kinase-dependent functions, p110β also has kinase-independent scaffolding functions, since some of the defects observed in p110β knockout cells are rescued by the expression of a kinase-dead mutants of p110β^8–10^.

The p110β subunit can regulate endocytosis and autophagy through its ability to bind to Rab5, a small GTPase with a key role in protein trafficking^11^. The p110β isoform, and not p110α, can uniquely bind to the active form of Rab5, Rab5-GTP, allowing for a coordinated regulation of PI3K activity and Rab5 activity^12,13^. The p85α subunit also binds directly to Rab5 and can downregulate Rab5 activity via p85-encoded GAP activity^14^. Consistent with this finding, knockdown of p85α resulted in increased Rab5-GTP levels^11^. In addition, p110β binding can protect Rab5-GTP from the GAP activity of p85α under conditions where growth factors are low^11^. Thus, p110β has been suggested to act as a sensor for growth factor levels and can induce autophagy by activating Rab5 signaling. Whether the role of p110β regulation of Rab5 changes during active receptor signaling has not yet been determined.

A previous report showed that there are at least two sites within p110β important for Rab5 binding^13^, one that includes the Ras-binding domain (RBD) of p110β within residues 136–270, and one that includes the helical domain of p110β within residues 658–759^13^. A subsequent study generated two point mutations within the helical domain, Q596C and I597S, which were sufficient to disrupt Rab5 binding^15^. This study set out to characterize the key residues within the p110β RBD that mediate binding to Rab5 and also the corresponding Rab5 residues critical for the binding of p110β. Mutation of these key residues allowed the generation of non-binding mutants of p110β and Rab5 that can be used to further study the role of this complex in the regulation of cell properties.

## Results

### Identification of p110 β binding surface on Rab5

**Figure 1:**
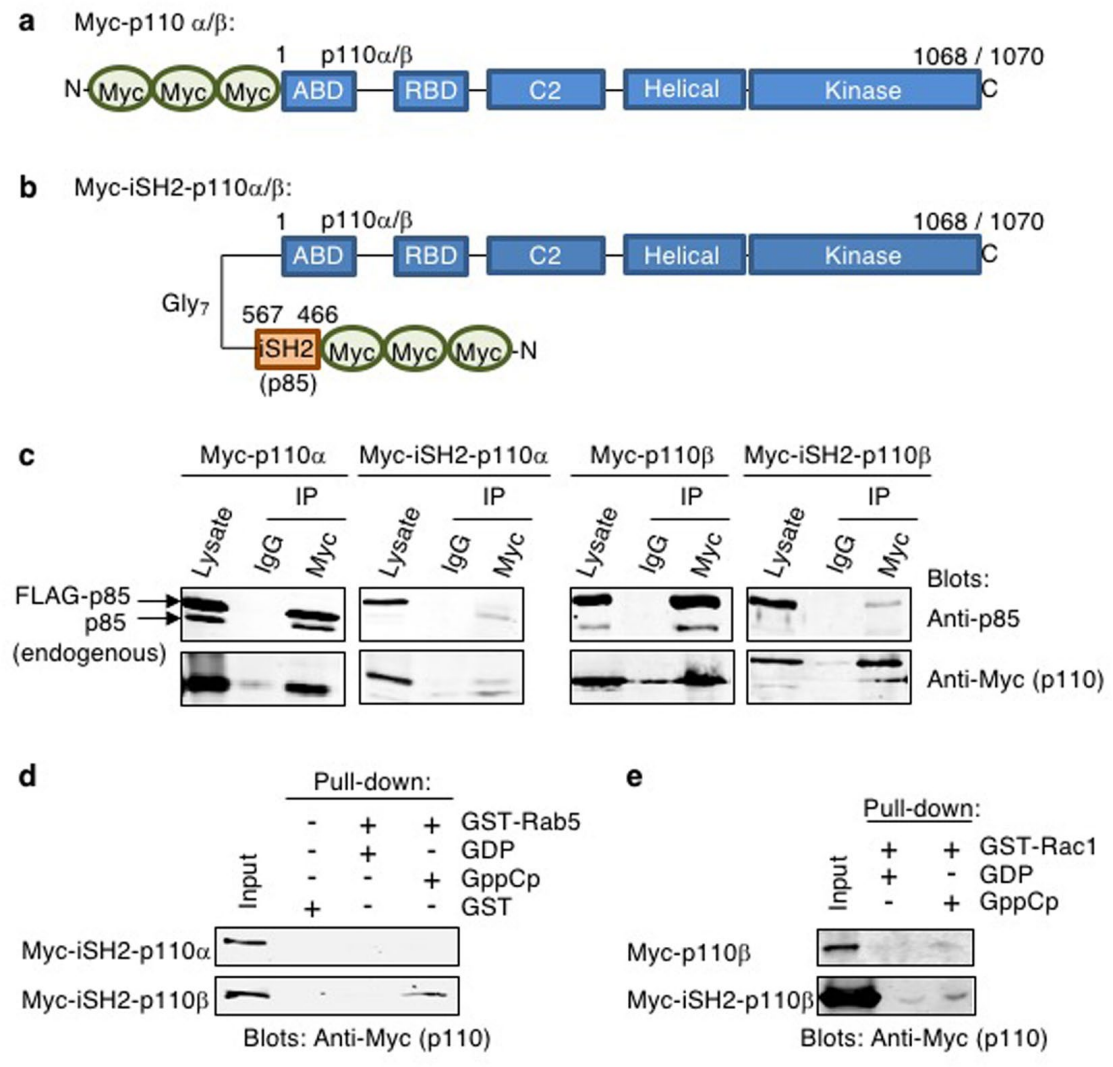
Chimeric Myc-iSH2-p110α and Myc-iSH2-p110β proteins do not associate well with p85 and p110β binds specifically to the active form of Rab5. (**a**) Schematic diagram of Myc-tagged p110α and Myc-p110β. (**b**) Schematic diagram of chimeric Myc-iSH2-p110 proteins in which the iSH2 region of p85α has been linked onto the N-terminus of the p110 proteins via a flexible glycine linker. (**c**) COS-1 cells were co-transfected with FLAG-p85 and Myc-p110 or Myc-iSH2-p110, as indicated. Cell lysates were immunoprecipitated with control mouse IgG or Myc antibody, resolved by SDS-PAGE with control lysates and immunoblotted as indicated. Full-length images of the cropped blots are in Supplementary Fig. S2a. (**d**) COS-1 cells were transfected with the indicated Myc-iSH2-p110 protein and lysates were used in a pull-down analysis. GST or GST-Rab5 was loaded with GDP or GppCp (a non-hydrolysable analogue of GTP) as indicated and lysates containing Myc-iSH2-p110 proteins were allowed to bind. Bound proteins were detected by immunoblotting with an anti-Myc antibody. The input lane corresponds to 4% of the total. Full-length images of the cropped blots are in Supplementary Fig. S2b. (**e**) GST-Rac1 was loaded with the indicated nucleotide and allowed to bind Myc-p110β or Myc-iSH2-p110β proteins from transfected COS-1 cell lysates. Bound Myc-tagged proteins were detected by immunoblotting with anti-Myc antibody. Input lanes contain 5% of the total protein used in each pull-down sample. Full-length images of the cropped blots are in Supplementary Fig. S2c.

The objective of this study was to map critical residues within the RBD of p110β and the Rab5 protein by assessing the binding of engineered mutants of each protein. The p110β protein is typically co-expressed with additional p85 protein, to bind and stabilize it^6,11^. However, since the BH domain of p85 encodes Rab5 GAP activity and can also bind Rab5, the p85:Rab5 interaction could interfere with the analysis of mutant p110β and/or Rab5 proteins. Therefore, a chimeric p110β protein was generated, modeled after an earlier version by Hu *et al*., in which the iSH2 domain of p85 was linked to the N-terminus of p110β with a flexible glycine linker, but with an N-terminal triple Myc tag^16^ (Fig. 1a,b). A co-immunoprecipitation analysis was used to assess amount of associated endogenous or ectopically expressed p85 to the Myc-iSH2-p110β protein, and the analogous Myc-iSH2-p110α protein. Both associated with little endogenous p85 as compared to the corresponding Myc-p110α/β proteins lacking the iSH2 regions (Fig. 1c). Therefore, this large N-terminal tag serves to both stabilize the p110β protein and also reduces the association with endogenous or ectopically expressed p85^16^. It was also confirmed that the Myc-iSH2-p110β protein, and not Myc-iSH2-p110α, retained its specificity for binding to Rab5-GTP^12^ using a pull-down analysis with a non-hydrolysable GTP analogue, GppCp (Fig. 1d). Since p110β has also been reported to bind to Rac1 via its Ras-binding domain (RBD), we also verified that the Myc-iSH2-p110β protein could bind to the activated form of Rac1 (Fig. 1e)^17^. Therefore the stabilized form of p110β, Myc-iSH2-p110β, was shown to be suitable for mapping key residues within Rab5 and p110β needed to mediate their interaction.

**Figure 2:**
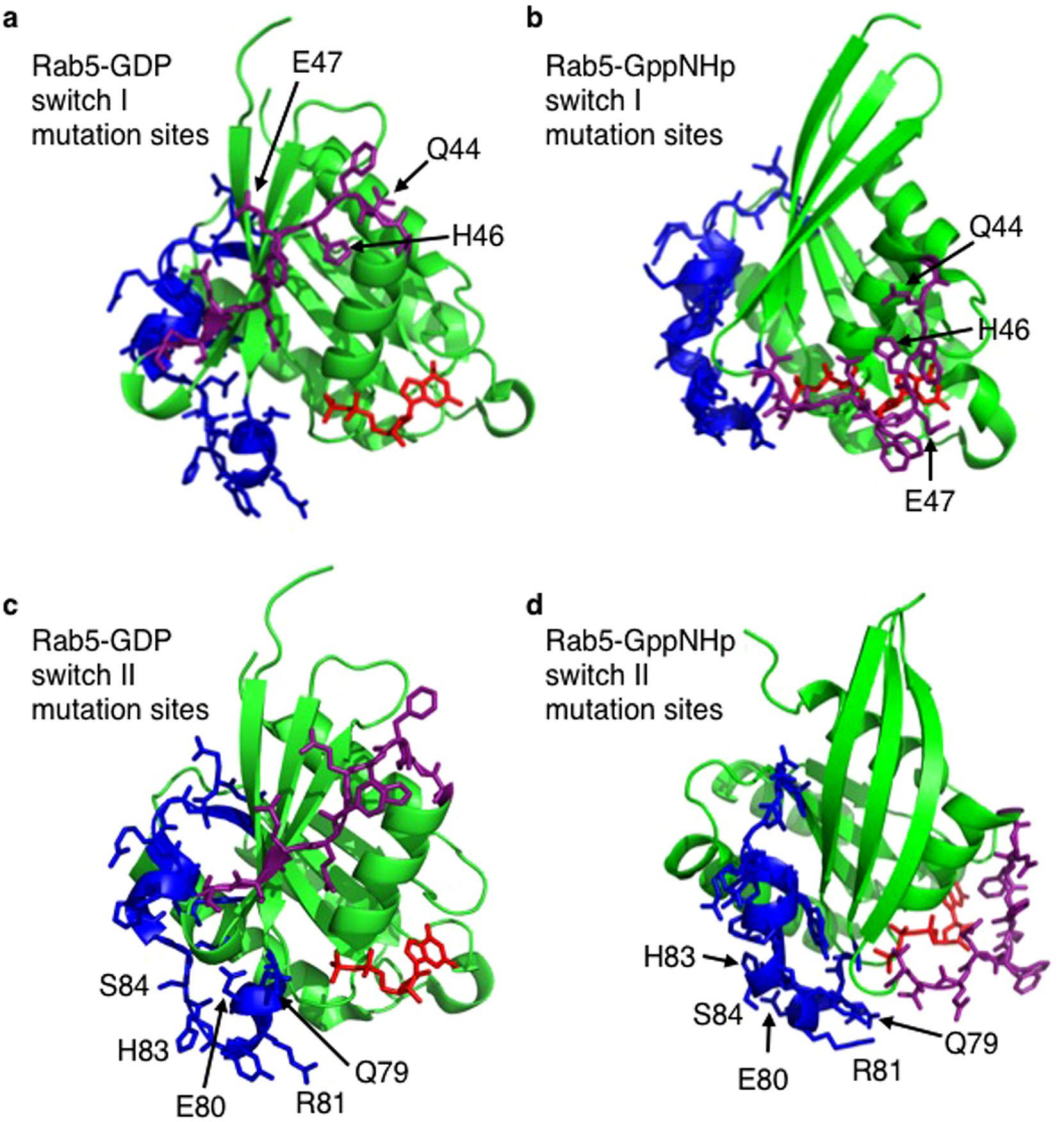
Location of the Rab5 mutations within switch I and switch II regions. Crystal structures of Rab5 in both the GDP-bound (**a**,**c**) and GppNHp-bound (**b**,**d**) conformations, where GppNHp is a non-hydrolysable analogue of GTP. Bound nucleotides are shown in red. Switch I is purple and moves away from the nucleotide in the GDP-bound conformation. Switch II is blue. The switch I region mutation sites are shown within Rab5-GDP (**a**; 1TU4) and within Rab5-GNP (**b**; 1R2Q), whereas the switch II region mutations are shown within Rab5-GDP (**c**; 1TU4) and within Rab5-GNP (**d**; 1R2Q).

**Figure 3:**
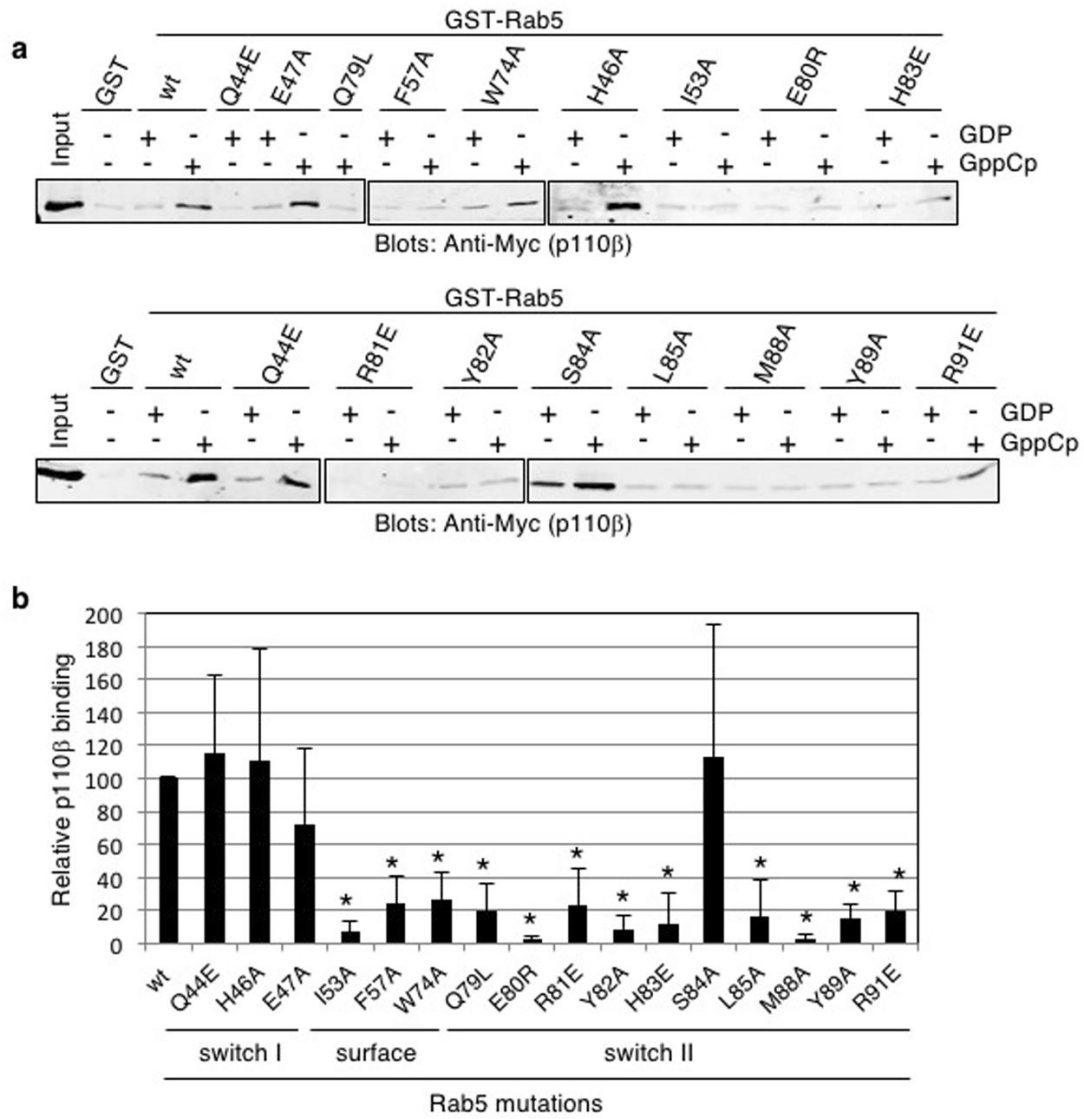
Myc-iSH2-p110β does not bind to Rab5 switch II region mutants. (**a**) GST, GST-Rab5 wild-type (wt) and mutant (as indicated) fusion proteins (10 µg) immobilized on glutathione Sepharose beads were loaded with either GDP or GppCp and incubated with Myc-iSH2-p110β from transfected COS-1 lysates. Bound p110β was detected using an immunoblot analysis with an anti-Myc antibody. Full-length images of the cropped blots are in Supplementary Fig. S3. (**b**) Quantification of pull-down binding data from at least 3 independent experiments with results similar to panel a. Intensity was measured in arbitrary units and normalized to that of Rab5-wt-GppCp; mean ± SD. *P < 0.01, as compared to p110β binding to wild-type Rab5-GppCp, using a series of paired, two-tailed t-tests.

GTPases like Rab5 exist in two distinct conformations when bound to GDP and GTP with the most substantial structural alterations occurring in two regions known as switch I (amino acids 43–52) and switch II (amino acids 77–95)^18^ (Fig. 2). Since p110β binds selectively to Rab5-GTP and not Rab5-GDP, it was reasoned that the switch regions of Rab5 were likely key for interactions with p110β. Therefore, amino acids in the Rab5 switch regions were the focus of the mutational analysis and testing for binding of p110β. Additional criteria included the accessibility of the residue in the GppNHp (a non-hydrolysable GTP-analogue)-bound crystal structure and that they adopt a different conformation in the GDP crystal (Fig. 2). The residues characterized included: Q44, H46, and E47 in switch I and Q79, E80, R81, Y82, H83, S84, L85, M88, Y89, R91 in switch II, as well as nearby surface residues I53, F57 and W74. Some were mutated to alanine and others to a residue of opposite charge in an attempt to disrupt p110β binding. Rab5-Q79L is a well-characterized mutation that lacks GTPase activity and locks Rab5 in an active GTP-bound conformation^19^. Pull-down experiments were conducted using wild-type and mutant GST-Rab5 loaded with GDP or GppCp, and an input of Myc-iSH2-p110β lysate (Fig. 3). Little or no binding of Myc-iSH2-p110β was observed to a negative control GST protein.

**Figure 4:**
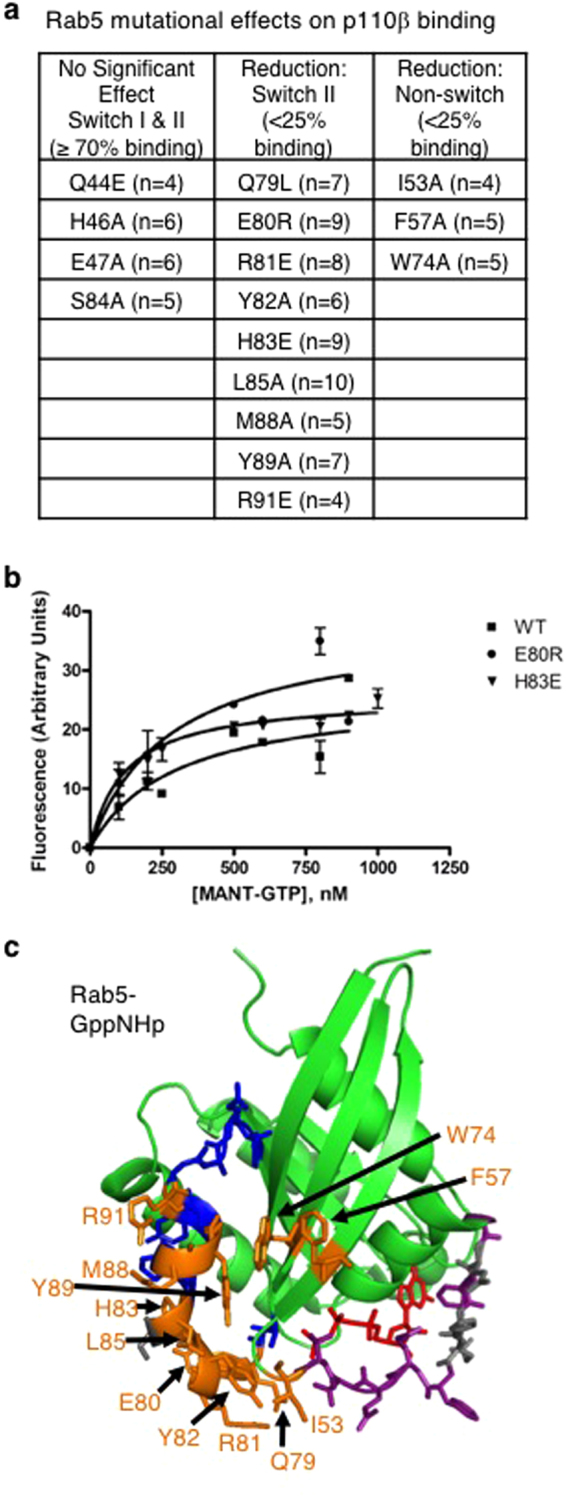
Rab5 mutants that affect p110β binding yet still bind nucleotide. (**a**) Summary of Rab5 mutations organised by their effect on Myc-iSH2-p110β binding. Experiments were carried out with different Rab5 mutant combinations so the number of replicates (n) for each mutant is indicated in brackets. (**b**) A MANT-GTP fluorescent nucleotide binding assay was performed with selected Rab5 mutants that do not bind p110β. Titration of 1 μM Rab5 wild type (WT), E80R and H83E mutants with increasing concentrations of MANT-GTP were carried out as detailed in the methods. (**c**) Rab5-GppNHp structure (1R2Q) modified from Fig. 2d displaying the mutated amino acids that had no significant effect on p110β binding (grey), or significantly reduced binding (orange).

A series of independent, paired, two-tailed t-test was conducted to compare Myc-iSH2-p110β binding in normalized wild-type GST-Rab5 and mutant GST-Rab5 conditions. Mutations in the switch I region, Q44E, H46A and E47A did not significantly affect Myc-iSH2-p110β binding to Rab5 (Figs 3 and 4a). In contrast, all mutations within the switch II region, except S84A, significantly reduced Myc-iSH2-p110β binding, and so did the mutations in three nearby residues, I53A, F57A and W74A.

To determine if these Rab5 mutations disrupted the overall folding of this relatively small protein, selected mutants (E80R and H83E) were purified (Supplementary Fig. S1) and tested in functional assays to determine their binding affinity for fluorescent GTP (Fig. 4b)^20,21^. The K_D_ values for MANT-GTP binding were 300 nM for wild type Rab5, 280 nM for Rab5-E80R and 120 nM for Rab5-H83E. These results show that both Rab5 mutants analyzed bind GTP with similar or higher affinity than does wild type Rab5 suggesting that the mutations do not impact p110β interactions due to a reduction in GTP-binding affinity. In addition, some of these Rab5 mutations (Q79L) or different alterations of these same residues (F57Y, R81A, H83A) have previously been shown to retain GTP-binding or Rab5 function^22^. These data defined the p110β binding surface on Rab5 (Fig. 4c), mainly within switch II.

### Identification of a Rab5 binding site in the RBD of p110β

**Figure 5:**
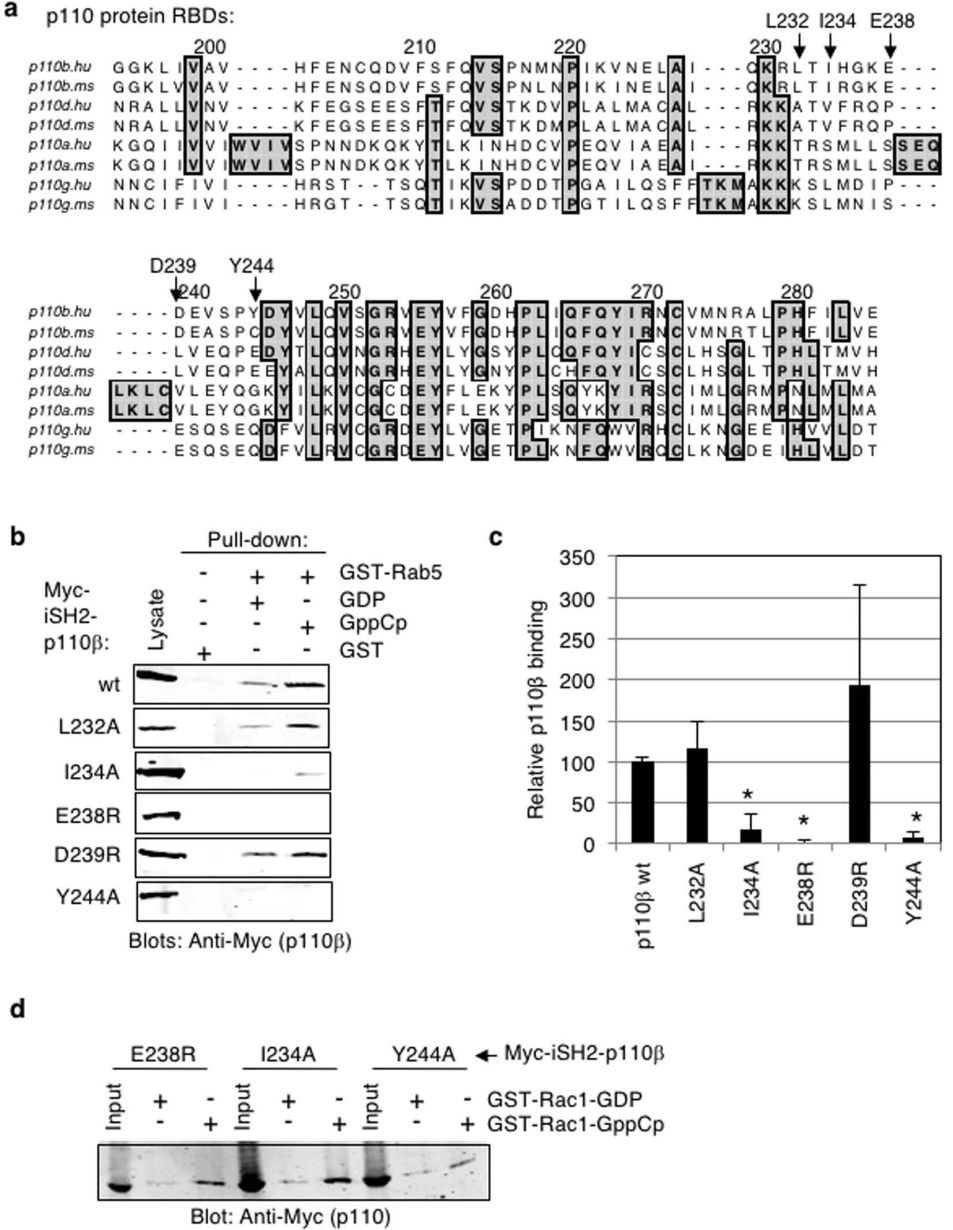
Identification of a Rab5 binding site within the RBD of p110β. (**a**) Sequence alignment of Ras-Binding Domain (RBD) of p110β (p110b; amino acids 194–285 in human and 188–279 in mouse), p110δ (p110d; amino acids 187–278), p110α (p110a; amino acids 187–289) and p110γ (p110g; amino acids 217–309) from human (hu) and mouse (ms) using multiple sequence alignment software, T-Coffee (MacVector). Shaded boxes indicate conserved residues. Numbering is for p110β residues. Arrows indicate residues that were chosen for mutation. (**b**) Pull-down assay where GST-Rab5 was loaded with either GDP or GppCp and incubated with each Myc-iSH2-p110β mutant (as indicated) and analyzed as before. Blots are representative of 3 independent binding experiments. Full-length images of the cropped blots are in Supplementary Fig. S4a-b. (**c**) Quantification of pull-down binding data from panel b and replicates; intensity measured in arbitrary units and normalized to wild-type Myc-iSH2-p110β binding to Rab5GppCp; mean ± SD from 3 independent experiments. *P < 0.01, as compared to wild-type p110β binding to Rab5-GppCp, using a series of paired, two-tailed t-tests. (**d**) GST-Rac1 proteins loaded with the indicated nucleotides were used in pull-down experiments with the various Myc-iSH2-p110β mutant proteins. Bound proteins were detected by immunoblotting with anti-Myc antibodies. Input lanes contain 2% of the total protein used in each pull-down. Full-length images of the cropped blots are in Supplementary Fig. S4c.

**Figure 6:**
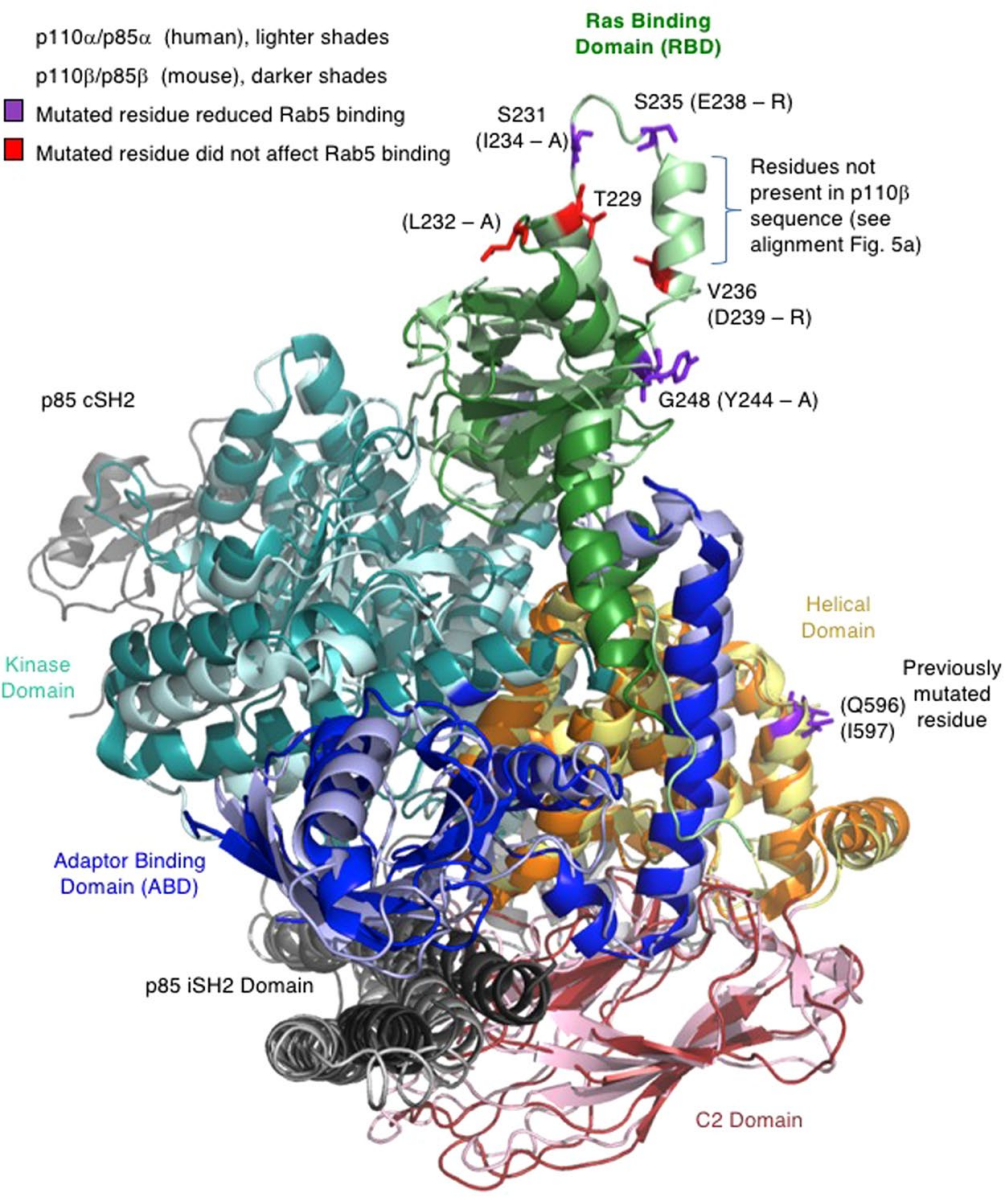
Structural alignment of p110β-cip85β and p110α-nip85α. Alignment of the wild-type p110β and p110α H1047R mutant full length protein crystal structures containing either the C-terminal SH2 and iSH2 domains of p85β or the N-terminal SH2 and iSH2 domains of p85α, respectively. Domains are indicated by color: ABD in blue, RBD in green, C2 domain in red/pink, Helical domain in orange/yellow, Kinase domain in teal and p85 domains in black/grey. The p110β-cip85β complex (PDBID: 2Y3A) is represented by dark colors; the p110α-nip85α (PDBID: 3HIZ) is represented by lighter shades of the same colors. *(upper)* Ras binding domain with mutated amino acids indicated in either red (no effect on Rab5 binding) or purple (reduction of Rab5 binding). p110α residues and numbers are indicated, p110β residues and numbers are shown in parentheses and dashes indicate the mutation characterized by this study.

A previous study used a deletion analysis of p110β to show that it contained at least two separate regions required for Rab5 binding, one between amino acids 136–270 that included portions of the adapter-binding domain (ABD) and RBD (residues 194–295), and a second region between amino acids 658–759 that included the helical and kinase domains^13^. Subsequent analyses focused on the helical domain of p110β and found that point mutations p110β-Q596C and p110β-I597S were each sufficient to disrupt binding to Rab5^11,15^. This study therefore focused on further defining the Rab5 binding site within the region of p110β containing the RBD, using a similar mutational analysis of p110β. Previous studies have shown that p110α has an RBD (amino acids 187–289) through which it interacts with the small GTPase Ras. Considering that Rab5 is also a small GTPase and has some structural similarity to Ras^18,23–25^, it was reasoned that it may bind p110β via its RBD. To determine the precise amino acids involved in Rab5 binding, sequence and structural alignments between the RBDs of p110α (which does not bind Rab5-GTP) and p110β (which binds Rab5-GTP) were performed (Figs 5a and 6).

The crystal structure of p110β has been solved in complex with the p85 cSH2 and iSH2 domains (PDB: 2Y3A) but the p110β amino acids 234 to 240 were disordered and therefore not visible in the structure^26^ (Fig. 6). Alignment of the amino acid sequences of the p110α and p110β (amino acids 194–285) RBDs demonstrated marked divergence between them (Fig. 5a). Despite this, the residues surrounding the disordered region overlay well in the structural alignment (Fig. 6). Five amino acids in the p110β RBD were identified to be distinct in both identity and positioning from p110α (in parentheses): L232 (T), I234 (S), E238 (S), D239 (V) and Y244 (G) though no conformational information was available for I234, E238 or D239. These amino acids were conserved between human and mouse p110β but were located within a region of the RBD poorly conserved between p110α, p110δ and p110γ (Fig. 5a). Each of these five amino acids were mutated individually within the full-length Myc-iSH2-p110β protein and then tested for Rab5 binding.

Pull-down experiments were performed with wild-type GST-Rab5 in either GDP or GppCp bound states (Fig. 5b,c). A paired, two-tailed t-test was conducted to compare GST-Rab5-GppCp binding in normalized wild-type Myc-iSH2-p110β and mutant Myc-iSH2-p110β conditions. The p110β mutants D239R and L232A showed Rab5-GppCp binding comparable to that of the wild-type p110β protein. The p110β-I234A, p110β-E238R and p110β-Y244A mutants showed significantly reduced binding to Rab5. These results suggest that p110β residues I234, E238 and Y244, but not L232 or D239, within the RBD of p110β contribute to Rab5 binding. Each of the p110β mutants that were defective for Rab5 binding was found to retain binding to Rac1 (Fig. 5d). This suggests the overall folding of the RBD of p110β was not greatly disrupted by these single point mutations and that different p110β residues are important for mediating binding to Rab5 as compared to Rac1. The location of the p110β residues within the RBD is indicated relative to those previously identified within the helical domain of p110β, Q596 and I597 (Fig. 6).

## Discussion

Mutations of Rab5 that reduced p110β binding were I53A, F57A, W74A, Q79L, E80R, R81E, Y82A, H83E, L85A, M88A, Y89A and R91E. Interestingly, S84A did not affect binding despite its location in the center of the binding site. The hydroxyl group of S84 may not make polar contacts with p110β, but the backbone of the protein might still be involved in binding and would not have been affected by its mutation to alanine. Most of the mutated residues that reduced p110β binding were located in the switch II region of Rab5. Three residues from the “inter-switch” region (or the sequence between the switch I and switch II regions *i.e*. AA 53–76) were also involved in the binding interface: I53, F57, and W74. Rab5 switch I shows large structural changes between GDP- and GTP-bound states as compared to switch II (Fig. 2), suggesting the Rab5-GTP-specific binding p110β relies on the more subtle structural alterations seen in switch II for its specificity.

G-protein effectors are known to bind selectively to the GTP-bound form and Rab5 has several known effector proteins including: Rabenosyn5, EEA1, Rapaptin5, APPL1 and p110β^12,27–30^. Many other proteins that complex with Rab5 have been identified by high-throughput screening using affinity-capture mass spectrometry^31,32^ and co-fractionation^33^ but their binding domains have not been characterized. The data available for the known Rab5 effector binding sites is summarized in Table 1 and compared to the results of the binding studies with p110β.

**Table 1:** Rab5 amino acids involved in binding effectors.

Amino acid involved in binding (Y/N)	Rabenosyn-5 (Rab22 crystal structure & mutation)	EEA-1 (crystal structure)	Rabaptin-5 (crystal structure & mutation)	APPL1 (mutation)	p110β (mutation)
Q20	Y	N	N	—	—
K22	N	Y	N	—	—
L38	N	N	N	N	—
K42	Y	N	N	Y	—
Q44	N	N	N	Y	N
H46	N	N	N	Y	N
E47	N	N	N	—	Y/N
F48	N	N	N	Y	—
Q49	N	Y	N	N	—
E50	Y/N	Y	N	N	—
S51	Y	Y	N	—	—
T52	N	Y	N	—	—
I53	Y	Y	N	N	Y
G54	Y	Y	Y	—	—
A55	Y/N	Y	Y	—	—
A56	Y	Y	N	—	—
F57	Y	Y	Y	N	Y
L58	N	N	Y	—	—
T59	Y/N	N	Y	—	—
Q60	N	N	N	—	—
K70	Y	N	N	—	—
E72	Y/N	Y	N	—	—
W74	Y	Y	Y	Y	Y
Q79	N	N	N	Y	Y
**E80**	**N**	**N**	**N**	**—**	**Y**
R81	Y	N	Y	—	Y
Y82	Y	Y	Y	—	Y
**H83**	**N**	**N**	**N**	**—**	**Y**
S84	Y	Y	N	—	N
L85	Y	Y	Y	Y	Y
P87	N	N	N	—	—
M88	Y	Y	Y	Y	Y
Y89	Y	Y	Y	Y	Y
R91	Y	Y	N	—	Y

Rab5 amino acids involved in binding with at least one of 5 effectors: Rabenosyn-5 (Eathiraj *et al*.^27^), EEA-1 (Mishra *et al*.^28^), Rabaptin-5 (Zhu *et al*.^18^), APPL1 (Zhu *et al*.^30^) and p110β are indicated in the left column. If mutation of the amino acid (mutation) affected binding to the effector or if the crystal structure revealed a contact point with that amino acid, a Y signifies “yes” or important for binding. If the mutation did not affect binding, or the crystal structure showed no interaction with that amino acid, a N depicts “no” or not important for binding. Y/N means both positive and negative results have been demonstrated. “-” means the amino acid has not been tested for binding. The amino acids unique to binding p110β are highlighted in bold.

Co-crystal structures that included Rab5 and effector were used to determine residues involved in binding to EEA1^28^ and Rabaptin-5^29^. Both EEA1 and Rabenosyn5 bound Rab5 via a FYVE zinc-finger motif. In contrast, the binding site for APPL1^30^ and Rabenosyn-5^27^ were determined using mutational binding assays, as this study has done for p110β. Rab5 bound to the PH domain of APPL1 in the context of APPL1 BAR-domain:PH-domain dimers. Similarly, Rab5 interacted with the coiled-coil C-terminal domains of Rabaptin-5 dimers. The RBD of p110β was demonstrated previously to be the binding site for Rab5^13^. Thus, Rab5 can interact with a variety of difference sequence motifs or domains.

The binding site of p110β on Rab5 included a majority of residues also involved in binding other Rab5 effectors (Table 1). Positioning of functional groups is important for effector recognition of their Rab binding partner. Residues F57, W74 and Y89 form an the invariant hydrophobic triad since they show significant conformation differences between Rab proteins while still being highly conserved in all Rab protein sequences^34^. In addition there are several other Rab5 residues including Y82, L85 and M88 that are important for Rab5 binding with many different effectors.

**Figure 7:**
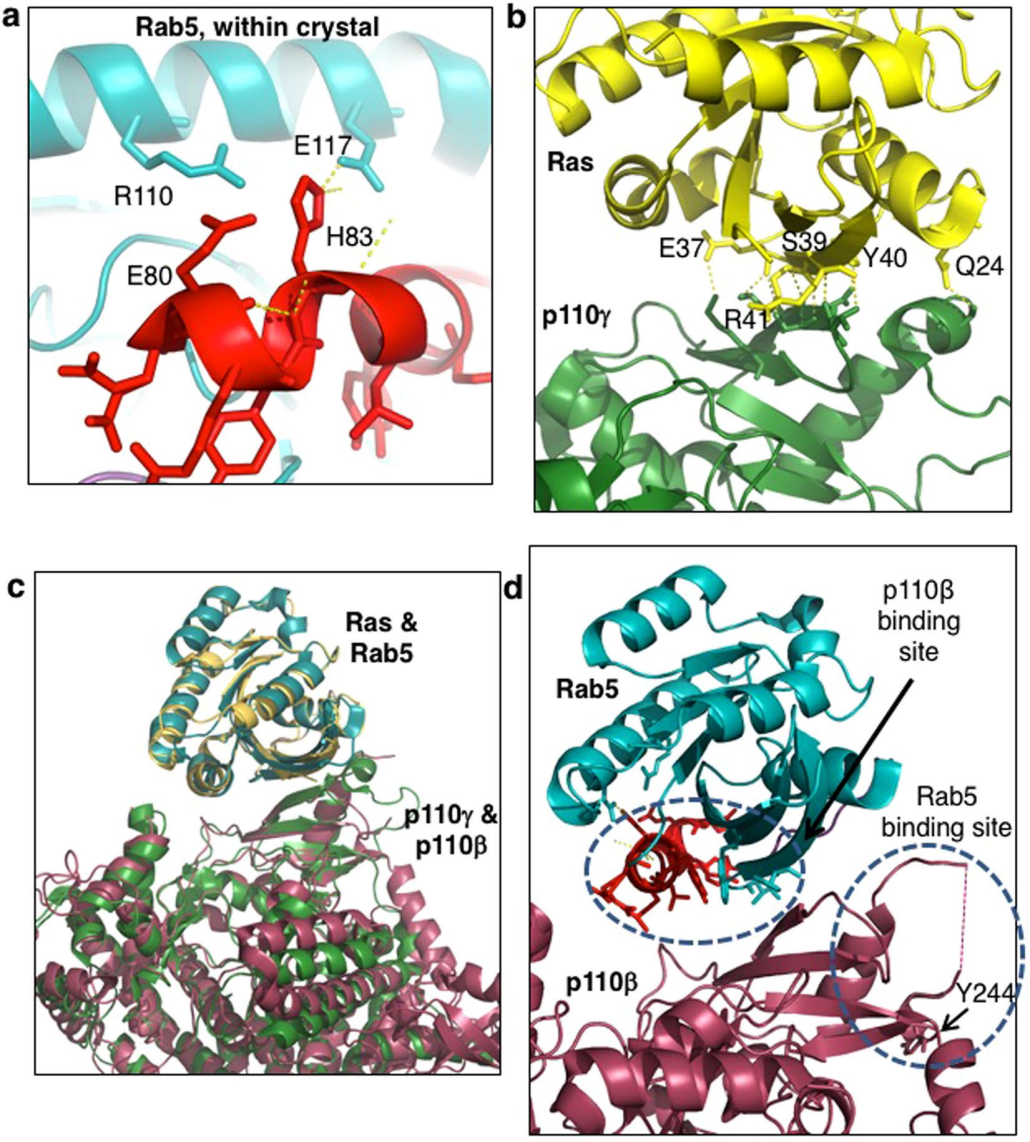
Structural alignment of H-Ras/Rab5 and p110γ/p110β illustrates differences in binding interfaces. (**a**) Close up of Rab5 intrachain hydrogen bonding between H83 and E116. Proximity of E80 and R110 should be noted as their side-chains may repel each other in the E80R mutant. (**b**) Close up of H-Ras:p110γ interface with H-Ras residues involved in polar contacts represented. H-Ras in yellow; p110γ in green. (**c**) Structural alignment overlay of the H-Ras:p110γ complex (1HE8) with Rab5 (1R2Q) and p110β (2Y3A) crystals. H-Ras in yellow; Rab5 in teal; p110γ in green; p110β in pink. (**d**) Close up of Rab5 and p110β interface. Residues involved in binding according to pull-down assay are shown as stick models and indicated by dashed circles. Rab5 in teal; p110β in pink; switch I ribbon in purple (not visible); switch II ribbon in red.

Rab5 residues unique to binding p110β were E80 and H83. These residues were mutated to a residue of opposite charge, and therefore their effect on p110β binding was substantial. In the wild-type Rab5-GNP crystal structure (1R2Q) H83 forms a hydrogen bond with E117 on α-helix 3 of Rab5 (Fig. 7a), so it is possible that the H83E mutation could be destabilizing to the Rab5 switch II loop because of charge repulsion between it and E117, causing displacement of many residues. Similarly, though E80 does not make intrachain contacts with its functional group, substitution of this residue to arginine may cause some charge repulsion from Rab5 residue R110, which is also positioned on α-helix 3 and very near to E80 in the crystal structure (Fig. 7a). The results of the MANT-GTP assay demonstrate that the E80R mutant and H83E mutant both retain the ability to bind GTP, which suggests proper folding and binding functions. It is interesting to note that an E80D mutation of Rab5 was discovered in a wide screen of lung adenocarcinoma cells, though it was not a statistically recurrent mutation^35^. Mutation of the identified Rab5 residues could provide a useful experimental approach to selectively disrupt p110β interactions and study their functional consequences in cells.

To pursue the Rab5 binding site within the RBD of p110β, this study focused on a flexible loop that was poorly conserved between p110α (that doesn’t bind Rab5) and p110β, a region of p110β that was disordered in the crystal structure^26^. E238R and Y244A had the largest reduction of binding to Rab5-GppCp, whereas I234A reduced binding to Rab5-GppCp by about 70%. Residues that did not affect binding to Rab5 were L232A and D239R, despite their proximity to I234 and E238, respectively.

Although there is no structure for the Rab5:p110β complex, there is a related co-crystal structure for H-Ras:p110γ^36^. H-Ras residues within switch I (Q25, D33, I36, E37, D38, S39, Y40, R41) and switch II (E63, Y64, R73) interact with the RBD of p110γ (Fig. 7b). p110γ residues F221, K223, T228, S230, E231, T232, K234, K251, K255, K256, S257 and L258 are similarly involved in H-Ras binding^36^. Of these p110γ residues, individual mutation of several residues was sufficient to prevent or reduce H-Ras binding (F221, T232, K234, K251, K255, K256).

Since Rab5 is highly related to H-Ras, a structural overlay of the Rab5 structure onto that of H-Ras was generated, and similarly the p110β structure was overlaid onto that of p110γ (Fig. 7c). Unlike for H-Ras where the switch I region was the most important for p110γ binding, for Rab5 mainly the switch II region (Q79L, E80R, R81E, Y82A, H83E, L85A, M88A, Y89A, R91E) and a small part of the interswitch region (I53A, F57A, W74A) were key for p110β binding. As well, the results of this study suggest that p110β residues in the flexible loop region of the RBD (I234, E238, Y244) rather than the residues in beta sheets of the RBD (like p110γ binding to H-Ras) are important for Rab5 binding. Therefore, though p110β bound Rab5 via its RBD, the interaction mechanism may be different from p110γ binding to H-Ras.

A recent study mutated residues in mouse p110β (S205D and K224A; the corresponding human p110β residues are S211 and K230) that were analogous to p110α and p110γ residues found to be important for Ras binding^17^. Although p110β does not bind Ras, mutation of these two residues was sufficient to prevent p110β binding to Rac1-GTP and Cdc42-GTP. They also showed that the p110β-S205D + K224A mutant retained binding to Rab5-GTP. Since we also find that p110β RBD mutants defective for Rab5 binding (E238R, I234A, Y244A) retain binding to Rac1-GTP, these combined results strongly suggest that Rab5 binds to p110β via a distinct mechanism as compared to Rac1 and Cdc42. In addition, the associated p85 protein contributed to Rab5-GTP binding, since expression of a Δp85 mutant lacking the SH3, BH and cSH2 domains resulted in decreased Rab5-GTP-bound p110β. Our results are consistent with these findings and suggest that Rab5 interacts with the p110β RBD via a distinct mechanism.

It is interesting to note that the Rab5-Q79L mutant that lacks GTPase activity, and maintains a constitutively active conformation Rab5-Q79L-GTP, does not bind p110β. Previous studies have shown Rab5-Q79L binds to p110β/p85 heterodimers^13^ and also to p85α alone^14^, suggesting that these interactions are primarily mediated through binding to p85.

The p110β binding site on Rab5 shares many residues with effectors and GAP proteins alike. Therefore, it is important to determine whether p110β is in fact a regulator of Rab5 or an effector. Because of the GAP activity of p85, it was expected that p110β may be involved in Rab5 deactivation by virtue of being bound to p85. However, the catalytic activity of p110β provides PI3,4,5P_3_, an important precursor in the generation of the PI3P on early endosomes, making it an effector of Rab5^37^. It was proposed that p110β engages the switch regions of Rab5 in order to stabilize the transition state and drive the hydrolysis of GTP. The binding sites between Rab5 and p110β have been identified and involved mainly residues from switch II of Rab5. It is unknown if the p110β-RBD:Rab5-switch II contacts stabilize the transition state of Rab5.

The mechanism of PI3K regulation of Rab5 is not yet fully understood. As a protein whose gene is often mutated in cancer, the function of wild-type p85α is of great interest^38–41^. The GAP function of p85^14^ theoretically limits the time and/or amount of homotypic early endosomal fusion by deactivation of Rab5 and therefore affects the proper trafficking of activated receptors^42^. The interaction between p110β and Rab5-GTP may be important for the generation of the lipid product PI3,4,5P_3_ on early endosomes, though p110β is recruited by the phosphotyrosine residues of RTKs through the p85 SH2 domain interactions and not by Rab-GTP. A non-catalytic function of p110β^8,9^ may be the p110β:Rab5-GTP switch region stabilization which is important for inactivation of Rab5 and early endosome development. In this report, residues on both Rab5 and p110β that are involved in their interaction were identified. The importance of the Rab5-GTP:p110β interaction may be elucidated through the characterization of these non-binding mutants in future cell-based functional experiments.

## Methods

### Plasmids and mutagenesis

The coding sequence for canine Rab5A (residues 2–215; Zerial lab) was amplified by PCR and subcloned into the *Bam*HI and *Eco*RI sites of pGEX6P1 vector (Amersham). The Myc_3_-p110α and FLAG-p85α plasmids have been described previously^14^. An insert encoding full-length wild-type human p110β (residues 1–1070; Backer lab) was amplified by PCR and subcloned into a *Bgl*II-*Eco*RI-digested pMyc_3_ vector^14^ to generate a Myc_3_-p110β plasmid. Both of the Myc_3_-p110α and Myc_3_-p110β plasmids were also further modified to add coding sequences for a seven residue glycine linker and the iSH2 region of p85α to generate Myc_3_-iSH2-p110α and Myc_3_-iSH2-p110β plasmids. Briefly, the glycine linker region was added using two complementary phosphorylated oligonucleotides encoding seven glycine residues, with flanking *Nhe*I sticky ends. This was cloned into the *Nhe*I sites within Myc_3_-p110α and Myc_3_-p110β so that only the most 5‘ *Nhe*I site was present after ligation. This remaining *Nhe*I site was then used to insert the PCR-amplified coding region for the iSH2 domain (residues 466–567) of bovine p85α. All plasmid inserts were verified by DNA sequencing.

Site-directed mutagenesis to introduce single amino acid changes were carried out using the QuikChange method (Stratagene), according to the manufacturer’s directions. DNA sequencing of the entire coding regions ensured that no additional mutations had been introduced.

### Cell culture and transfections

COS-1 cells from the American Type Culture Collection (#CRL-1650, CRL-1658) were used in transient transfections to express FLAG-p85 and each of: Myc_3_-p110α, Myc_3_-p110β, Myc_3_-iSH2-p110α or Myc_3_-iSH2-p110β protein. Transfections were carried out using lipofectamine (Invitrogen) and X-tremeGENE 9 (Roche) according to the manufacturer’s directions. Cells were cultured according to ATCC recommendations for fewer than six months from the time of resuscitation. All cell lines were authenticated by the supplier (http://www.ATCC.org).

### Pull-down experiments, immunoprecipitations and immunoblots

GST-Rab5 and GST-Rac1 pull-down binding experiments, immunoprecipitations and immunoblotting were carried out as previously described^14^. GST-Rab5 or GST-Rac1 complexes were loaded with either GDP (Sigma-Aldrich) or a non-hydroyzable analogue of GTP, GppCp (Jena Bioscience), as indicated. Most antibodies were purchased from Santa Cruz Biotechnology: mouse IgG agarose conjugate (sc-2342AC), anti-Myc agarose conjugate (sc-14428AC), anti-Myc (sc-789 and sc-40), with the exception of anti-p85 (Millipore; #05-217). Secondary antibodies were goat anti-mouse or goat anti-rabbit conjugated to infrared dye (LI-COR Biosciences; Lincoln, NB) and were visualized using LI-COR imaging and Odyssey software V3.0.

### MANT-GTP assay

GST-Rab5-bound glutathione Sepharose beads were incubated in a buffer containing EDTA (50 mM Tris-HCl pH 7.4, 150 mM NaCl, 1 mM dithiothritol, 10 mM EDTA pH 8.0) for 30 minutes at 4 °C to remove bound nucleotide. The Rab5 protein was cleaved from GST by PreScission protease (GE Healthcare, 27-0843-01) overnight at 4 °C in a buffer containing magnesium (50 mM Tris-HCl pH 7.4, 150 mM NaCl, 1 mM dithiothritol, 10 mM MgCl_2_). Cleaved Rab5 was collected and concentrated to 1 mL before being loaded onto a glutathione Sepharose HR16/10 column using the ÄKTApurifier liquid chromatography system and UNICORN 5.31 software (both from GE Healthcare) to remove any contaminating GST and PreScission protease. The unbound protein fractions were buffer exchanged into HEPES-buffered saline (25 mM HEPES 7.4, 150 mM NaCl, 10 mM MgCl_2_) and further purified by gel filtration chromatography using a Superdex200 Increase column (GE Healthcare). Protein concentration was determined by Lowry assay (Sigma-Aldrich).

The fluorescence binding assay was carried out in a black-walled 96-well plate. Varying concentrations of MANT-GTP (Molecular Probes, Cat# M-12415) were added to Rab5 (wild type or mutant protein, each 1 µM) in buffer (25 mM HEPES 7.4, 150 mM NaCl, 10 mM MgCl_2_) to a final volume of 200 µL and incubated for 2 hours at 30 °C. Fluorescence measurements (Ex 355 nm; Em 449 nm; cut-off 435 nm) are reported as change in fluorescence from time 0, and the 0 nM MANT-GTP control was subtracted from each^20,21^. At least 3 independent experiments were carried out for each Rab5 wild type and mutant protein and graphed as mean ± SD^20,21^. Data were analyzed by nonlinear regression using Prism 4 software (GraphPad Prism 4.00, San Diego, CA) for curve fitting and K_D_ calculations.

### Quantification and Statistical Analyses

Blots were scanned directly using the LI-COR Odyssey Infrared Imager (LI-COR Biosciences). The bands visualized using Odyssey software V3.0 were quantified using arbitrary intensity units. GST negative control lane intensity was subtracted from all sample bands for that blot. Protein expression in Myc_3_-iSH2-p110-expressing cell lysates was normalized to the wild-type protein, which was assigned a value of 1. The amount of Myc_3_-iSH2-p110β bound to GST-Rab5 fusion proteins in each pull-down experiment was normalized to wild-type Rab5-GTPγS (100%). Data from three or more independent experiments were combined as mean ± SD, as indicated in each figure legend. P-values to assess statistically significant differences were obtained using Prism software (GraphPad Prism 4.00, San Diego, CA) using a paired two-tailed t-test.

### Structural Representations

Figures were generated using PyMOL software (The PyMOL Molecular Graphics System, Version 1.4.1 Shrödinger, LLC.).

### Availability of data and materials

The structural datasets analyzed during the current study are available in the RCSB depository:

- 1TU4 (http://www.rcsb.org/pdb/explore/explore.do?structureId = 1tu4),
- 1R2Q (http://www.rcsb.org/pdb/explore/explore.do?structureId = 1r2q),
- 2Y3A (http://www.rcsb.org/pdb/explore/explore.do?structureId = 2y3a),
- 3HIZ (http://www.rcsb.org/pdb/explore/explore.do?structureId = 3hiz),
- 1HE8 (http://www.rcsb.org/pdb/explore/explore.do?structureId = 1he8).

The protein interaction datasets will be available in the IntACT depository upon publication.

## Electronic supplementary material

Supplementary Information

## References

[1] Yuan TL, Cantley LC (2008). PI3K pathway alterations in cancer: variations on a theme. Oncogene.

[2] Agarwal R, Carey M, Hennessy B, Mills GB (2010). PI3K pathway-directed therapeutic strategies in cancer. Current opinion in investigational drugs.

[3] Vanhaesebroeck, B., Guillermet-Guibert, J., Graupera, M. & Bilanges, B. The emerging mechanisms of isoform-specific PI3K signalling. *Nat Rev Mol Cell Biol* **1**1, 329–341, doi:nrm2882 [pii] 10.1038/nrm2882 (2010). 10.1038/nrm2882 20379207

[4] Vogt PK (2010). Phosphatidylinositol 3-kinase: the oncoprotein. Curr Top Microbiol Immunol.

[5] Wong KK, Engelman JA, Cantley LC (2010). Targeting the PI3K signaling pathway in cancer. Curr Opin Genet Dev.

[6] Yu J (1998). Regulation of the p85/p110 phosphatidylinositol 3′-kinase: stabilization and inhibition of the p110alpha catalytic subunit by the p85 regulatory subunit. Mol Cell Biol.

[7] Backer JM (2010). The regulation of class IA PI 3-kinases by inter-subunit interactions. Curr Top Microbiol Immunol.

[8] Ciraolo, E. *et al*. Phosphoinositide 3-kinase p110beta activity: key role in metabolism and mammary gland cancer but not development. *Sci Signal* **1**, ra3, doi:1/36/ra3 [pii] 10.1126/scisignal.1161577 (2008). 10.1126/scisignal.1161577 PMC2694958 18780892

[9] Jia, S. *et al*. Essential roles of PI(3)K-p110beta in cell growth, metabolism and tumorigenesis. *Nature* **454**, 776–779, doi:nature07091 [pii] 10.1038/nature07091 (2008). 10.1038/nature07091 PMC2750091 18594509

[10] Dou Z (2010). The class IA phosphatidylinositol 3-kinase p110-beta subunit is a positive regulator of autophagy. J Cell Biol.

[11] Dou Z (2013). Class IA PI3K p110beta subunit promotes autophagy through Rab5 small GTPase in response to growth factor limitation. Mol Cell.

[12] Christoforidis S (1999). Phosphatidylinositol-3-OH kinases are Rab5 effectors. Nat Cell Biol.

[13] Kurosu H, Katada T (2001). Association of phosphatidylinositol 3-kinase composed of p110beta-catalytic and p85-regulatory subunits with the small GTPase Rab5. J Biochem.

[14] Chamberlain MD, Berry TR, Pastor MC, Anderson DH (2004). The p85alpha Subunit of Phosphatidylinositol 3’-Kinase Binds to and Stimulates the GTPase Activity of Rab Proteins. J Biol Chem.

[15] Salamon RS (2015). Identification of the Rab5 binding site in p110beta: assays for PI3Kbeta binding to Rab5. Methods in molecular biology.

[16] Hu Q, Klippel A, Muslin AJ, Fantl WJ, Williams LT (1995). Ras-dependent induction of cellular responses by constitutively active phosphatidylinositol-3 kinase. Science.

[17] Fritsch R (2013). RAS and RHO families of GTPases directly regulate distinct phosphoinositide 3-kinase isoforms. Cell.

[18] Terzyan S, Zhu G, Li G, Zhang XC (2004). Refinement of the structure of human Rab5a GTPase domain at 1.05 A resolution. Acta Crystallogr D Biol Crystallogr.

[19] Li G (1995). Evidence for phosphatidylinositol 3-kinase as a regulator of endocytosis via activation of Rab5. Proc Natl Acad Sci USA.

[20] Eccleston JF, Moore KJ, Brownbridge GG, Webb MR, Lowe PN (1991). Fluorescence approaches to the study of the p21ras GTPase mechanism. Biochem Soc Trans.

[21] Remmers AE, Posner R, Neubig RR (1994). Fluorescent guanine nucleotide analogs and G protein activation. J Biol Chem.

[22] Li G, Stahl PD (1993). Structure-function relationship of the small GTPase rab5. J Biol Chem.

[23] Vetter IR, Wittinghofer A (2001). The guanine nucleotide-binding switch in three dimensions. Science.

[24] Schweins T, Wittinghofer A (1994). GTP-binding proteins. Structures, interactions and relationships. Curr Biol.

[25] Zhu G (2003). High resolution crystal structures of human Rab5a and five mutants with substitutions in the catalytically important phosphate-binding loop. J Biol Chem.

[26] Zhang X (2011). Structure of lipid kinase p110beta/p85beta elucidates an unusual SH2-domain-mediated inhibitory mechanism. Mol Cell.

[27] Eathiraj S, Pan X, Ritacco C, Lambright DG (2005). Structural basis of family-wide Rab GTPase recognition by rabenosyn-5. Nature.

[28] Mishra A, Eathiraj S, Corvera S, Lambright DG (2010). Structural basis for Rab GTPase recognition and endosome tethering by the C2H2 zinc finger of Early Endosomal Autoantigen 1 (EEA1). Proc Natl Acad Sci USA.

[29] Zhu G (2004). Structural basis of Rab5-Rabaptin5 interaction in endocytosis. Nat Struct Mol Biol.

[30] Zhu G (2007). Structure of the APPL1 BAR-PH domain and characterization of its interaction with Rab5. EMBO J.

[31] Hein MY (2015). A human interactome in three quantitative dimensions organized by stoichiometries and abundances. Cell.

[32] Huttlin EL (2017). Architecture of the human interactome defines protein communities and disease networks. Nature.

[33] Wan C (2015). Panorama of ancient metazoan macromolecular complexes. Nature.

[34] Merithew E (2001). Structural plasticity of an invariant hydrophobic triad in the switch regions of Rab GTPases is a determinant of effector recognition. J Biol Chem.

[35] Imielinski M (2012). Mapping the hallmarks of lung adenocarcinoma with massively parallel sequencing. Cell.

[36] Pacold ME (2000). Crystal structure and functional analysis of Ras binding to its effector phosphoinositide 3-kinase gamma. Cell.

[37] Shin HW (2005). An enzymatic cascade of Rab5 effectors regulates phosphoinositide turnover in the endocytic pathway. J Cell Biol.

[38] Samuels Y, Waldman T (2010). Oncogenic mutations of PIK3CA in human cancers. Curr Top Microbiol Immunol.

[39] Cheung LW (2011). High Frequency of PIK3R1 and PIK3R2 Mutations in Endometrial Cancer Elucidates a Novel Mechanism for Regulation of PTEN Protein Stability. Cancer Discov.

[40] Sun M, Hillmann P, Hofmann BT, Hart JR, Vogt PK (2010). Cancer-derived mutations in the regulatory subunit p85alpha of phosphoinositide 3-kinase function through the catalytic subunit p110alpha. Proc Natl Acad Sci USA.

[41] Ross RL (2013). Identification of mutations in distinct regions of p85 alpha in urothelial cancer. PLoS ONE.

[42] Chamberlain MD (2008). Disrupted RabGAP function of the p85 subunit of phosphatidylinositol 3-kinase results in cell transformation. J Biol Chem.

