# Supplementary material for: Identification of the Binding Sites on Rab5 and p110beta Phosphatidylinositol 3-kinase
Source: Sci Rep. 2017 Nov 23;7:16194. doi: 10.1038/s41598-017-16029-6 (PMC5700975; doi:10.1038/s41598-017-16029-6)
Supplement: Supplementary file 1 — Supplementary Information [file 41598_2017_16029_MOESM1_ESM.pdf]

Supplementary Information

Identification of the Binding Sites on Rab5 and p110beta Phosphatidylinositol 3-kinase

Dielle E. Whitecross[1], and Deborah H. Anderson[1,2*]

[1]Cancer Research, Saskatchewan Cancer Agency and [2]Departments of Oncology and Biochemistry, College of Medicine, 107 Wiggins Road, University of Saskatchewan, Saskatoon, Saskatchewan, S7N 5E5, Canada

Supplementary figure legends

Figure S1

a) Gel filtration (Superdex200) column elution profiles for wild type (WT) Rab5 and Rab5 mutants, E80R and H83E.

b) Aliquots (0.5 μg) of purified wild type (WT) Rab5, Rab5-E80R and Rab5-H83E, both before and after gel filtration, were resolved by SDS-PAGE (15%) and stained with Coomassie blue.

Figure S2. Full-length images of the cropped blots presented in the main figures.

a) Full-length images of Figure 1c

b) Full-length images of Figure 1d

c) Full-length images of Figure 1e

Figure S3. Full-length images of the cropped blots presented in the main figures.

a) – e) Full-length images of Figure 3a

Figure S4. Full-length images of the cropped blots presented in the main figures.

a) Full-length images of Figure 5b

b) Full-length images of Figure 5b

c) Full-length images of Figure 5d

Supplemental Figure S1

a

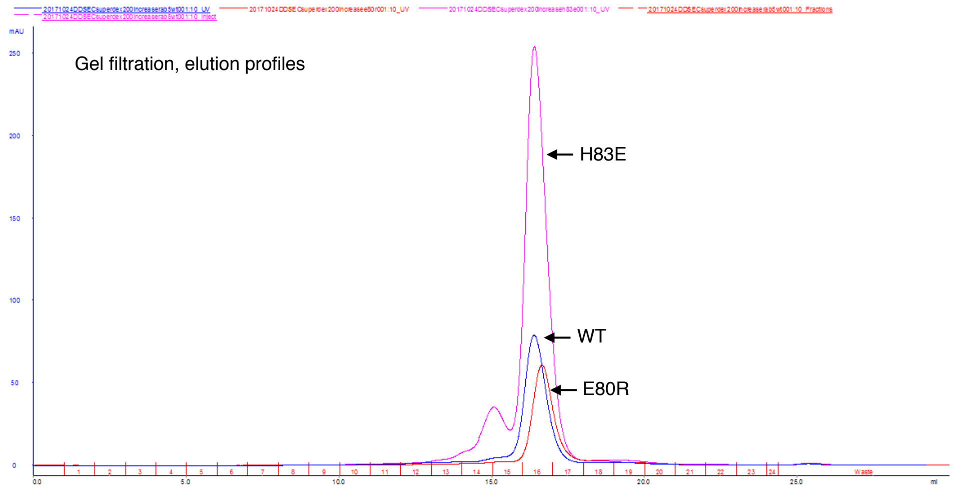

b

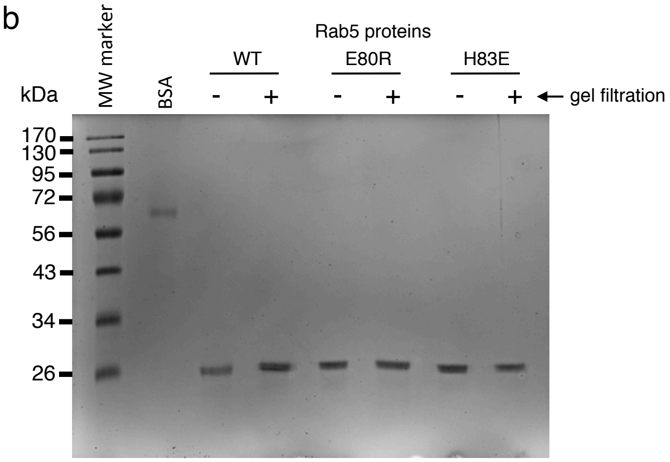

Coomassie Blue Stained SDS-PAGE

Supplemental Figure S2

Full-sized: Figure 1c

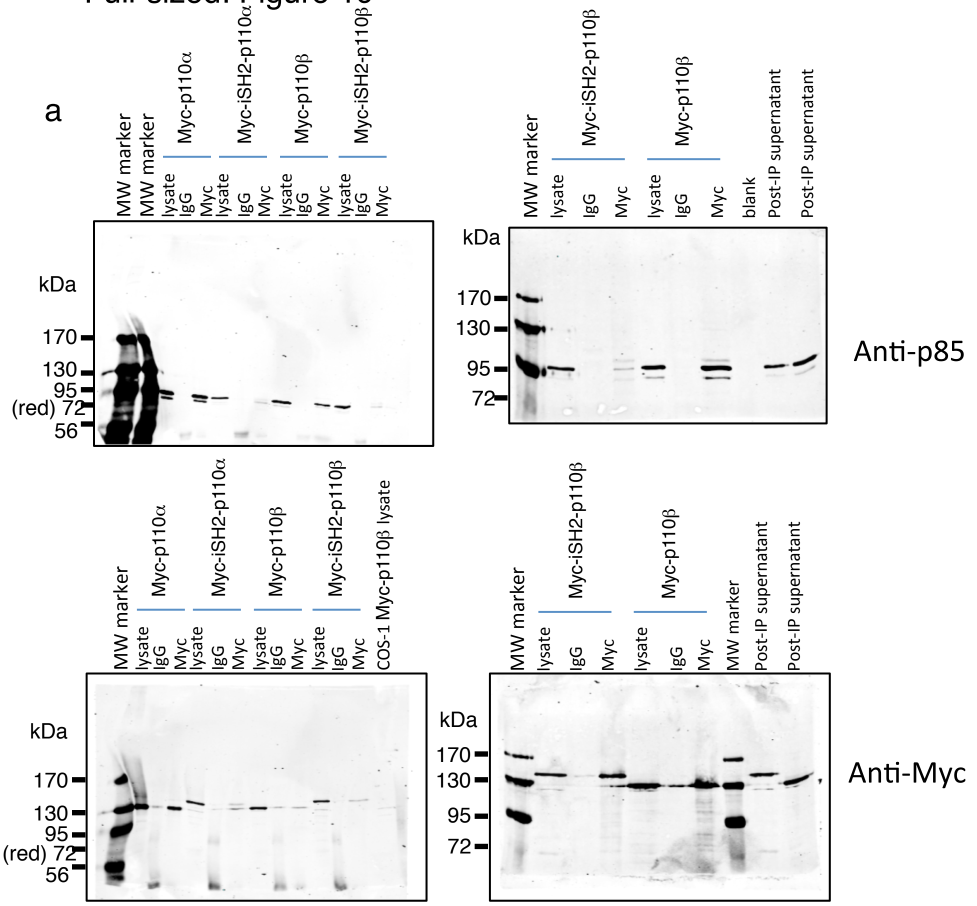

b Full-sized: Figure 1d

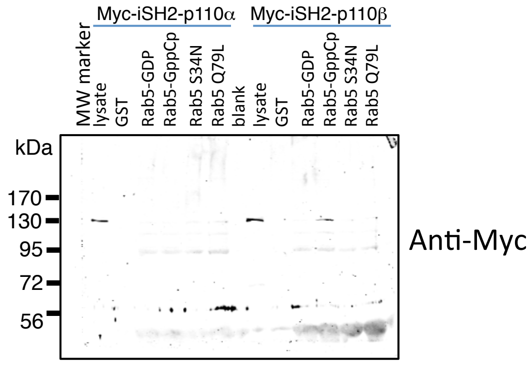

c Full-sized: Figure 1e

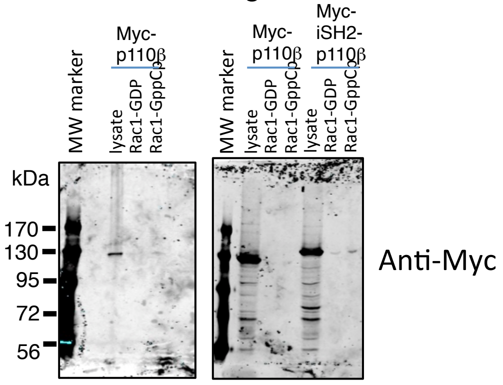

Supplemental Figure S3

Full-sized: Figure 3a

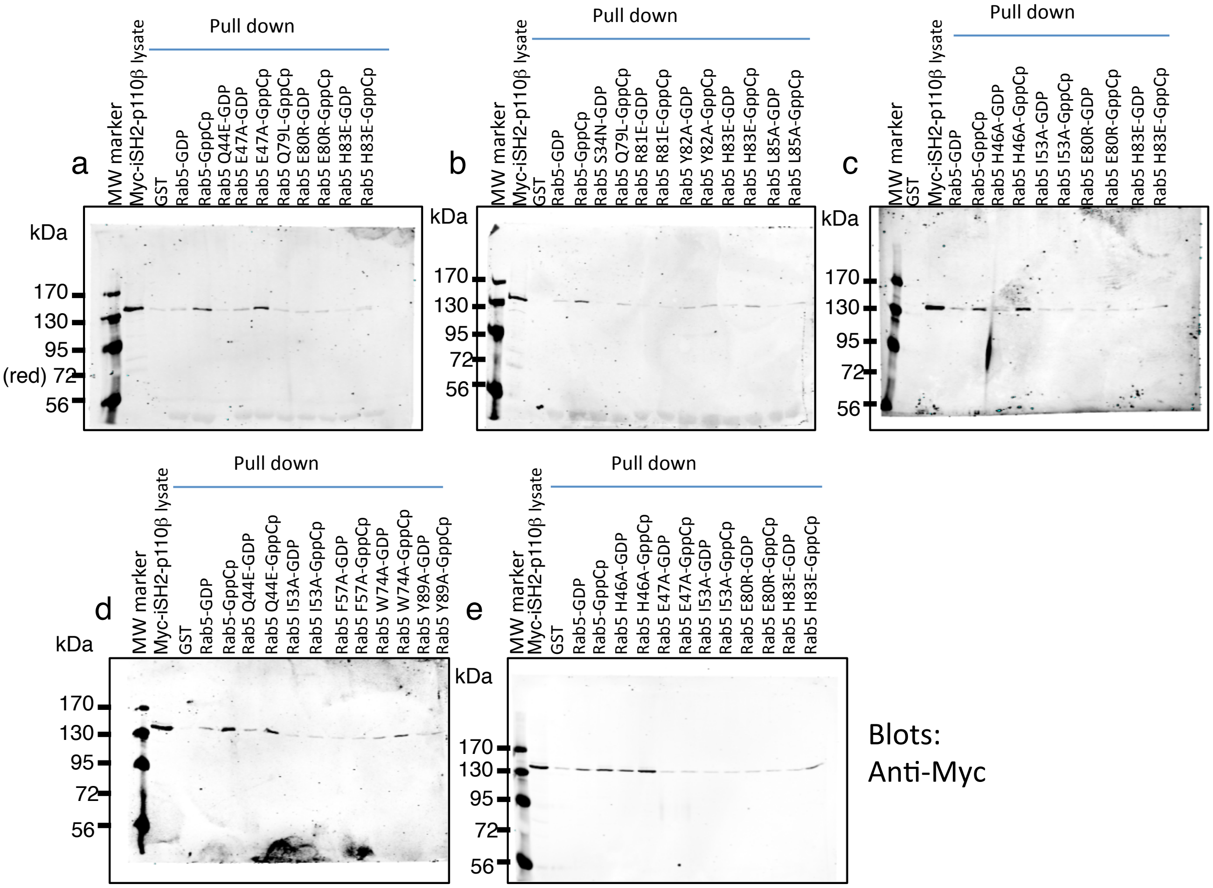

Blots:
Anti-Myc

Supplemental Figure S4

a

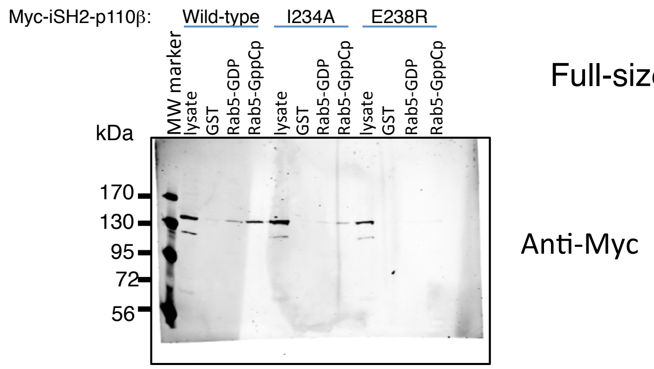

Full-sized: Figure 5b

b

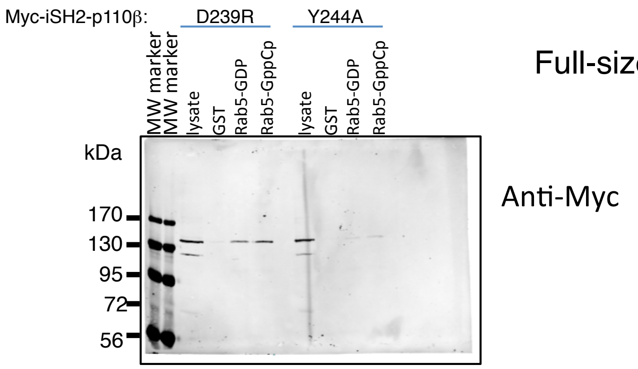

Full-sized: Figure 5b

c

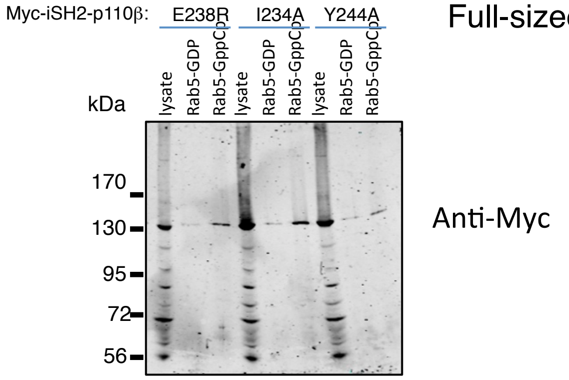

Full-sized: Figure 5d
